# SPERM FACTORS AND EGG ACTIVATION: PLCzeta as the sperm factor that activates eggs: 20 years on

**DOI:** 10.1530/REP-22-0148

**Published:** 2022-05-13

**Authors:** Karl Swann

**Affiliations:** 1 School of Biosciences, Cardiff University, The Sir Martin Evans Building, Cardiff, UK

One of the simplest and most significant questions that we can ask about fertilization is: how does the sperm activate the egg? It has been known since the 1970s that sperm activate the development of eggs by causing a transient increase in the cytosolic free Ca^2+^ ion concentration. These studies then shifted the question to one of how the sperm could cause the increases in Ca^2+^ in the egg. The publication of the discovery of phospholipase Czeta (PLCZ1) in August 2002 was a critical moment in our understanding of mammalian egg activation (Saunders *et al.* 2002). This was because it identified PLCZ1 as the protein in sperm extracts that could cause Ca^2+^ oscillations and mouse egg activation. It was the culmination of many years of searching to find the elusive soluble sperm factor that causes Ca^2+^ release in mammalian eggs. This factor is also often referred to as the ‘sperm-born oocyte-activating factor’ or SOAF. While some aspects of the search for sperm-derived egg activating factors are not over, there can be little doubt that PLCZ1 plays the central role in mammalian egg activation during *in vitro* fertilization (IVF) and after the widely used technique of intracytoplasmic sperm injection (ICSI). It represents the first identified and foremost sperm-derived egg activating factor.

The idea that the sperm contains some factor that activates the egg at fertilization can be traced back to Loeb’s work in the early 20th century (Loeb 1913). He proposed that the sperm introduces a substance (a ‘lysin’) into sea urchin eggs to cause the formation of the fertilization envelope. He also proposed that there was a second factor that was essential for the egg to develop and not undergo degeneration. It is difficult to map some of these ideas into modern thinking around the theories of signalling at fertilization developed over the last 50 years. Nevertheless, it was clear that the early pioneers of research on fertilization were trying to address the key issue of how the sperm, as a small cell, could provide a stimulus for the development of the much larger egg. The modern era for understanding how the sperm activates an egg followed the recognition that an increase in cytosolic Ca^2+^ concentrations is the key trigger of egg activation (Steinhardt *et al.* 1974, Ridgway *et al.* 1977, Fulton & Whittingham 1978). A Ca^2+^ increase during egg activation has been shown to be both necessary and sufficient to stimulate the development in all the species studied (Stricker 1999). Studies in the 1970s and 1980s of cytosolic Ca^2+^ were on eggs from sea urchins, frogs, fish, ascidians and mice or hamsters. The Ca^2+^ increases at fertilization can occur in the form of single Ca^2+^ waves, or as a series of Ca^2+^ oscillations, depending upon the species (Stricker 1999). There are now many more species where Ca^2+^ increases have been measured in eggs at fertilization and the central role of Ca^2+^ in physiological egg activation has been shown to be conserved. The predominant idea in the 1980s and early 1990s was that the sperm acts via a transmembrane receptor to stimulate G-proteins or tyrosine kinases to stimulate (inositol 1,4,5-trisphosphate (InsP_3_) production from phospholipase C (PLC) enzymes within the egg (Shilling *et al.* 1994). It was established that InsP_3_ causes Ca^2+^ release in eggs (Whitaker & Irvine 1984, Busa *et al.* 1985, Miyazaki 1988), but whether a sperm transmembrane receptor was involved was not clear. The idea of transmembrane receptors initiating Ca^2+^ has had some experimental support in frog eggs. However, despite the discovery of many molecules involved in sperm–egg binding in mammals, none have been found the link to InsP_3_ production of Ca^2+^ release. In contrast, it is clear from studies in mouse and sea urchin eggs that the sperm and egg undergo fusion for several seconds, or even a minute, before Ca^2+^ release is initiated (McCulloh & Chambers 1992, Lawrence *et al.* 1997). This suggested that the sperm could introduce some soluble (hydrophilic) factors that could diffuse into the egg to promote Ca^2+^ release.

The first direct evidence for a soluble sperm factor came from studies that showed that injecting sperm extracts into sea urchin eggs could trigger the formation of the fertilization envelope (Dale *et al.* 1985). It was not established whether this factor was either sperm specific or protein based. No one has reproduced this work despite the simplicity of the experiment, and the issue of soluble sperm factors in sea urchins remains unresolved. A few years later, mammalian sperm extracts were shown to trigger egg activation in mouse, rabbit and hamster eggs (Stice & Robl 1990, Swann 1990). The key observation was that mammalian sperm extracts could also trigger Ca^2+^ oscillations in hamster, mouse and human eggs (Swann 1990, 1994, Homa & Swann 1994). Demonstrating Ca^2+^ oscillations was significant because in mammals at least, the injection of many substances such as Ca^2+^ can cause egg activation, but only the sperm was known to trigger prolonged Ca^2+^ oscillations. Hence, by noting the presence of prolonged Ca^2+^ oscillations, a real ‘sperm factor’ can be distinguished from an artefact. This is one reason why I regard the term ‘SOAF’ as being of little more value than ‘sperm factor’ because SOAF fails to highlight the key issue of Ca^2+^ changes. It is the pattern of Ca^2+^ changes which can provide the hallmark of the physiological agent. The early observation that sperm extracts could trigger Ca^2+^ oscillations was later replicated and extended in mouse and cow eggs, as well as in eggs/oocytes from ascidians and nemertean worms (Stricker 1997, Wu *et al.* 1997, Kyozuka *et al.* 1998). In all these cases, it was confirmed that sperm contained a specific protein-based factor capable of causing the Ca^2+^ oscillations seen in eggs at fertilization. Some of the initial candidate sperm factor proteins did not stand up to further scrutiny, and the reproducibility of reports on the effects of sperm factor candidates in eggs has remained a problem for the field.

Shortly after researchers discovered evidence for a soluble sperm factor, Palermo and colleagues in Belgium started using ICSI as a new way to treat male factor infertility in humans (Palermo *et al.* 1992). Injecting the sperm was found to be highly effective in activating development, which was surprising given the predominant view was that sperm mediate Ca^2+^ release via plasma membrane receptors. However, the effectiveness of ICSI in itself did not prove that sperm contain an activating factor because it was possible to argue that the injection of the Ca^2+^ in the medium along with the sperm could be responsible for egg activation. Hence, ICSI may not use a physiological mechanism, but this turned out not to be the case. It was found that the injection of human sperm into human or mouse eggs leads to a series of Ca^2+^ oscillations that could not be mimicked by sham injections (Tesarik *et al.* 1994, Nakano *et al.* 1997). Hence, ICSI provided further evidence that sperm contain a factor that activates the egg via the cytoplasm and not via plasma membrane signalling. Subsequent work in mouse ICSI suggested that the factor or SOAF was tightly bound to the sperm head (Kimura *et al.* 1998). It was not clear at the time whether the rather insoluble SOAF that is active during mouse ICSI was the same as the soluble factor present in sperm extracts.

A key step came in 1998 when it was shown that mammalian sperm extracts contained a highly active PLC that could account for InsP_3_ production and Ca^2+^ oscillations in eggs (Jones *et al.* 1998). Hence, the sperm factor could be a PLC. Subsequent studies of the mammalian sperm PLC activity suggested that it was not due to one of the known isoforms. Then in 2002, my colleagues and I showed that the novel isoform PLCZ1 was the sperm protein in extracts that causes Ca^2+^ oscillations in mouse eggs (Saunders *et al.* 2002). We and others also found that PLCZ1 is present in sperm from different mammals, including humans, and that its ability to cause Ca^2+^ oscillations and the activation of early development was conserved (Cox *et al.* 2002, Rogers *et al.* 2004, Kurokawa *et al.* 2005, Yoon & Fissore 2007). It was noteworthy that within 2 years of the publication of our original paper it was shown that the ‘insoluble’ sperm-head-bound SOAF that is active in mouse ICSI is in fact PLCZ1 (Fujimoto *et al.* 2004). About a dozen different groups have now independently confirmed that PLCZ1 can cause Ca^2+^ oscillations in eggs and in each case the responses are similar to those induced by sperm (Ito *et al.* 2011, Sanders & Swann 2016). The reliability and reproducibility of the actions of PLCZ1 are in contrast to other candidate sperm factors that have either proved unreproducible by others or else have not been independently reproduced (Sanders & Swann 2016).

Since its discovery, PLCZ1 has been found in all mammalian genomes and detected in sperm of many different species. It also appears to be present in birds and some fish. This special issue contains a series of articles that bring our knowledge of PLCZ1 up to date. The paper by Thanassoulas *et al.* (2022) focuses on what we know about the structure of PLCZ1 and how it relates to its ability to cause Ca^2+^ oscillations in eggs. It is noted that we have a good understanding of the significance of the primary structure of PLCZ1 but we still have much to learn about proteins that may interact with PLCζ in the sperm or egg and how these proteins may affect PLCZ1 activity. We also still do not know how PLCZ1 localizes to intracellular membranes rather than the plasma membrane. Another paper by Gupta *et al.* (2022) relates the story of the development of ICSI and how we developed an understanding of the critical role of PLCZ1 in explaining why it works, and why it often fails in domestic animals. The paper by Satouh (2022) describes the experiments using PLCZ1 null sperm to fertilize mouse eggs. These experiments show that PLCZ1 alone accounts for all the Ca^2+^ oscillations after ICSI, but they also suggest that another mechanism may exist to promote Ca^2+^ oscillations in eggs in IVF. The nature of this mechanism, which takes about 40 min to act, remains unresolved. This provides context for another article by Iwao and Ueno (2022) which discusses possible non-PLCZ1 sperm factor candidates in newt eggs where physiological polyspermy occurs, and multiple sperm fusions are required to activate the eggs. We also have two articles by Cardona Barberán *et al.* (2022) and Jones *et al.* (2022) on the role of PLCZ1 in human fertility. Both papers describe the growing body of evidence that shows that PLCZ1 plays a critical role in human fertility and that a lack, or relative absence, of active PLCZ1 in human sperm is likely to result in poor rates of fertilization after ICSI. They offer their perspectives on the nature and extent of this problem, its diagnosis and how it could be addressed in clinical treatments. In the next 20 years, the assessment of PLCZ1 may become part of the standard andrology screen that men may receive in order to inform the treatment in IVF clinics. I hope that we will eventually be able to use the knowledge we have gained about PLCZ1 and Ca^2+^ release in eggs to offer patients facing fertilization failure with a simple and effective means to ensure egg activation during IVF treatment.

## Declaration of interest

The author declares that there is no conflict of interest that could be perceived as prejudicing the impartiality of this editorial.

## Funding

This work did not receive any specific grant from any funding agency in the public, commercial or not-for-profit sector.
